# MiR-497 downregulation contributes to the malignancy of pancreatic cancer and associates with a poor prognosis

**DOI:** 10.18632/oncotarget.2184

**Published:** 2014-07-08

**Authors:** Jianwei Xu, Tianxiao Wang, Zhe Cao, Hua Huang, Jian Li, Wenjing Liu, Shanglong Liu, Lei You, Li Zhou, Taiping Zhang, Yupei Zhao

**Affiliations:** ^1^ Department of General Surgery, Peking Union Medical College Hospital, Chinese Academy of Medical Sciences and Peking Union Medical College, Beijing 100730, China; ^2^ Key Laboratory of Carcinogenesis and Translational Research (Ministry of Education), Department of head and neck Surgery, Peking University Cancer Hospital & Institute, Beijing 100142, China

**Keywords:** pancreatic cancer, miRNA, chemoresistance, prognosis

## Abstract

Chemoresistance is one of the causes of poor prognosis in pancreatic cancer patients. However, the mechanisms of resistance remain unclear. Here we screened miRNAs associated with drug resistance in pancreatic cancer, and identified a panel of miRNAs dysregulated in gemcitabine-resistance pancreatic cancer cells, including 13 downregulated miRNAs and 20 upregulated miRNAs. Further studies focusing on miR-497 demonstrated that miR-497 suppressed cells proliferation, decreased the percentage of S phase cells, re-sensitized cells to gemcitabine and erlotinib, and attenuated migration and invasion capacities. Furthermore, fibroblast growth factor 2 and fibroblast growth factor receptor 1 were confirmed as miR-497 targets. Overexpression of miR-497 inhibited tumor growth *in vivo*. Additionally, miR-497 expression was significantly downregulated in pancreatic cancer tissues compared with tumor-adjacent samples (P=0.000). Low expression of miR-497 was an independent adverse prognostic factor of pancreatic cancer (P=0.01, hazard ratio=2.762, 95% confidence interval: 1.159–6.579). Together these results indicate that miR-497 could be a new therapeutic target and prognostic marker of pancreatic cancer.

## INTRODUCTION

Pancreatic cancer is a lethal disease, with a 5-year survival rate of less than 5% [1]. One of the reasons underlying poor prognosis of pancreatic cancer is drug resistance. Gemcitabine is a first line chemotherapeutic agent that is widely used for treatment of advanced pancreatic cancer. However, only 9.4% of patients with metastatic pancreatic cancers achieve partial response, and 34.5% show a progressive disease during the chemotherapy [2]. Newer therapeutic regimens and some targeted therapies, such as epidermal growth factor receptor tyrosine kinase inhibitor, are superior to conventional regimens, but the overall survival is still unsatisfactory [3, 4]. Therefore, determining the mechanisms of drug resistance and re-sensitizing pancreatic cancer cells to chemotherapy are essential measures for improving survival.

MiRNAs are involved in the regulation of carcinogenesis and drug resistance as oncogenes or tumor suppressors [5, 6]. Many studies have reported roles for miRNAs in the regulation of the progression of pancreatic cancer, including in tumor growth, metastasis and chemoresistance [7]. However, most studies only investigated a select panel of miRNAs. The profile of miRNAs associated with drug resistance in pancreatic cancer has not yet been identified.

The current study screened miRNAs associated with drug resistance in pancreatic cancer and identified a panel of miRNAs dysregulated in drug-resistant pancreatic cancer cells, including miR-497. We examined the role of miR-497 in pancreatic cancer cells and the expression levels of miR-497 in pancreatic cancer tissues and evaluated prognostic values.

## RESULTS

### Screening miRNAs associated with drug resistance in pancreatic cancer

**Figure 1:**
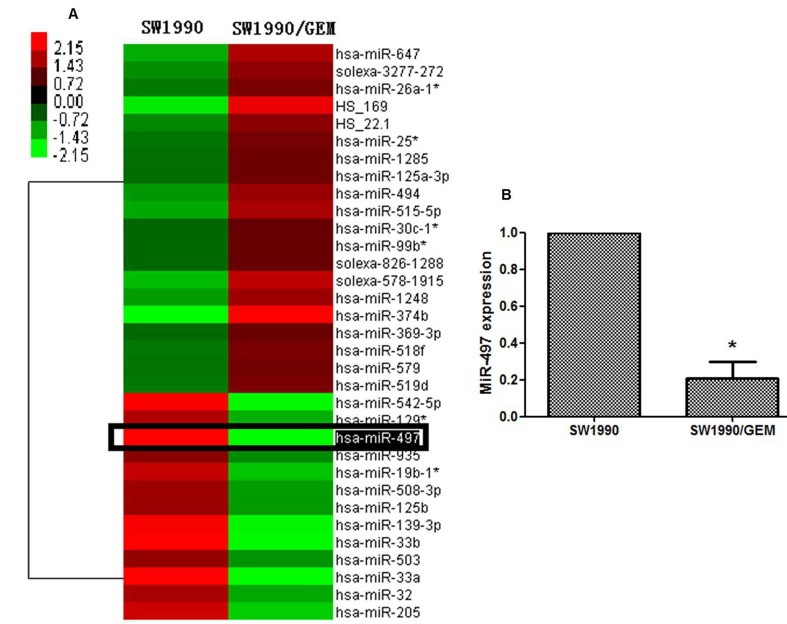
MiRNA profile associated with drug resistance in pancreatic cancer cells (A) The heat map shows 33 miRNAs whose expression is altered > 4- or < 0.25-fold in gemcitabine-resistant SW1990 cells (SW1990/GEM cells) compared with SW1990 cells. (B) Validation of miR-497 expression levels by qRT-PCR. U6 served as an internal control (*P < 0.05).

To identify miRNAs associated with drug resistance in pancreatic cancer, we screened the miRNA expression profile in parental SW1990 and gemcitabine-resistant SW1990 (SW1990/GEM) cells using Illumina miRNA arrays. The criteria for identification of miRNAs associated with drug resistance were fold change > 4 or < 0.25 and *P* < 0.05 (Student *t* tests). A total of 33 miRNAs out of 1146 miRNAs probes in the Illumina microarray platform were identified, including 13 downregulated miRNAs and 20 upregulated miRNAs (Figure 1A). Among these miRNAs, miR-497 was chosen for further investigation based on the following reasons. First, miR-497 showed the largest change in expression level in SW1990/GEM cells (0.00794-fold change). Quantitative reverse-transcription polymerase chain reaction (RT-PCR) further showed that the expression level of miR-497 in SW1990/GEM cells was significantly downregulated (Figure 1B). Second, miR-497 was previously reported to be involved in regulating chemosensitivity in colorectal cancer [8].

### MiR-497 suppressed pancreatic ductal adenocarcinoma (PDAC) cell proliferation

We first confirmed the expression levels of miR-497 after transfection of mimics or inhibitor in SW1990 cells. As expected, miR-497 levels were significantly upregulated or downregulated after transfection of mimics or inhibitor, respectively (Figure S1).

**Figure 2:**
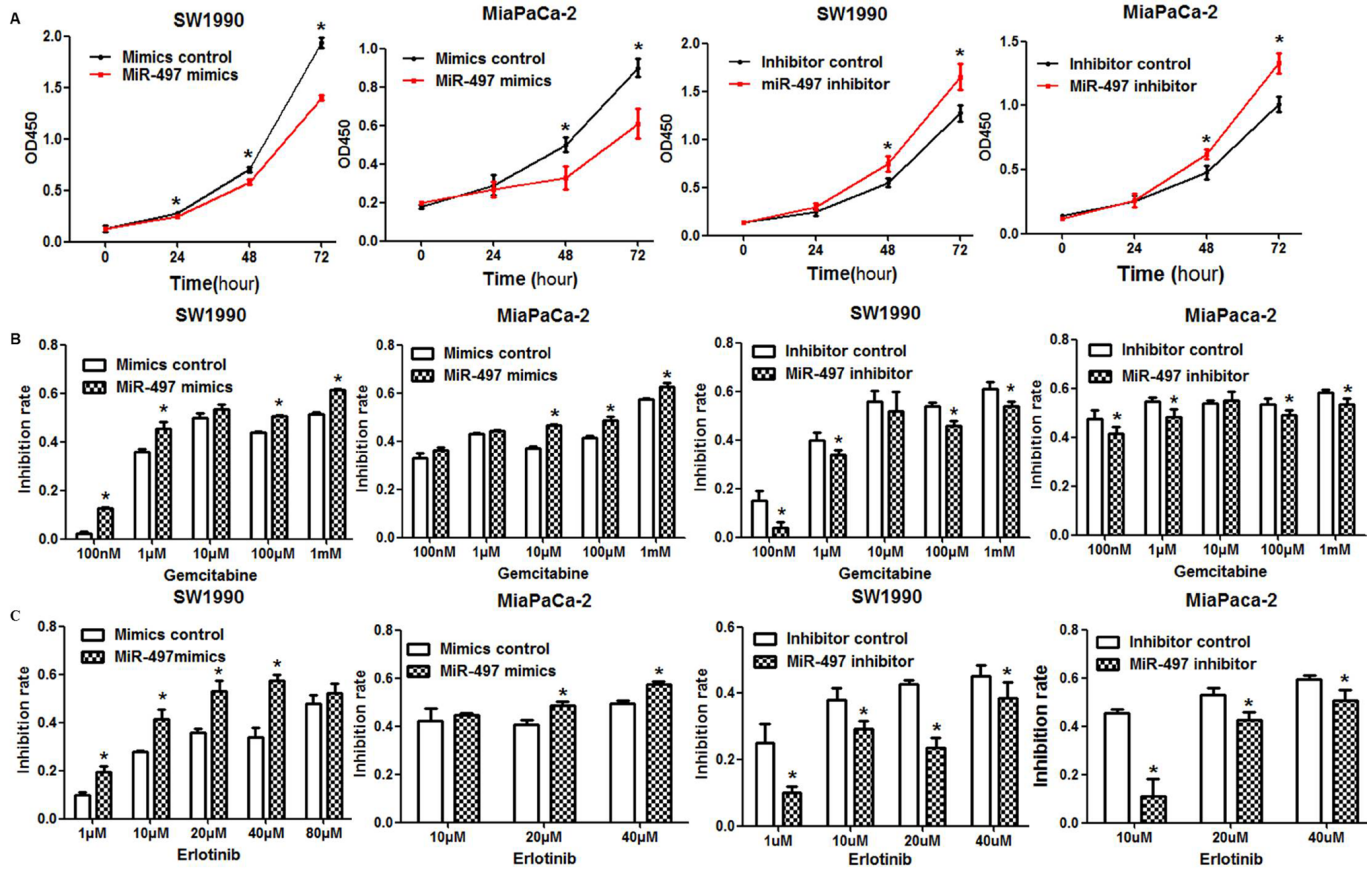
Effects of miR-497 on proliferation and chemosensitivity (A) PDAC cells were transfected as indicated, and proliferation was analyzed by CCK8 assay. The optical density at the wavelength of 450 nm (OD450) was positively associated with the count of viable cells (*P < 0.05). (B) Transfected cells were treated with different doses of gemcitabine for 48 h. Cell viability was evaluated using the CCK8 assay, and the inhibition rate of each dose was calculated. (C) The sensitivity of PDAC cells to erlotinib was examined. Data are displayed as the mean ± SD (*P < 0.05).

CCK-8 assays were performed to assess the effects of miR-497 mimics or inhibitor on the proliferation of SW1990 and MiaPaCa-2 PDAC cells. We observed a significantly decreased growth rate in miR-497 upregulated cells compared with control cells in both PDAC cell lines (Figure 2A). In addition, downregulation of miR-497 by inhibitor promoted the proliferation of both PDAC cell lines (Figure 2A).

### MiR-497 re-sensitized PDAC cells to gemcitabine and erlotinib

We next examined the effects of miR-497 levels on chemosensitivity of PDAC cells to gemcitabine and erlotinib. Upregulation of miR-497 in SW1990 and MiaPaCa-2 cells by transfection with mimics significantly increased the inhibitory effects of gemcitabine treatment compared with cells transfected with mimics control (Figure 2B). In contrast, PDAC cells transfected with inhibitor were more resistant to gemcitabine compared with cells transfected with inhibitor control (Figure 2B). Consistent with these results, upregulation of miR-497 increased PDAC cell sensitivity to erlotinib (Figure 2C), while decreased sensitivity to erlotinib was observed in cells downregulated for miR-497 (Figure 2C).

### MiR-497 induced cell cycle arrest in PDAC cell lines

**Figure 3:**
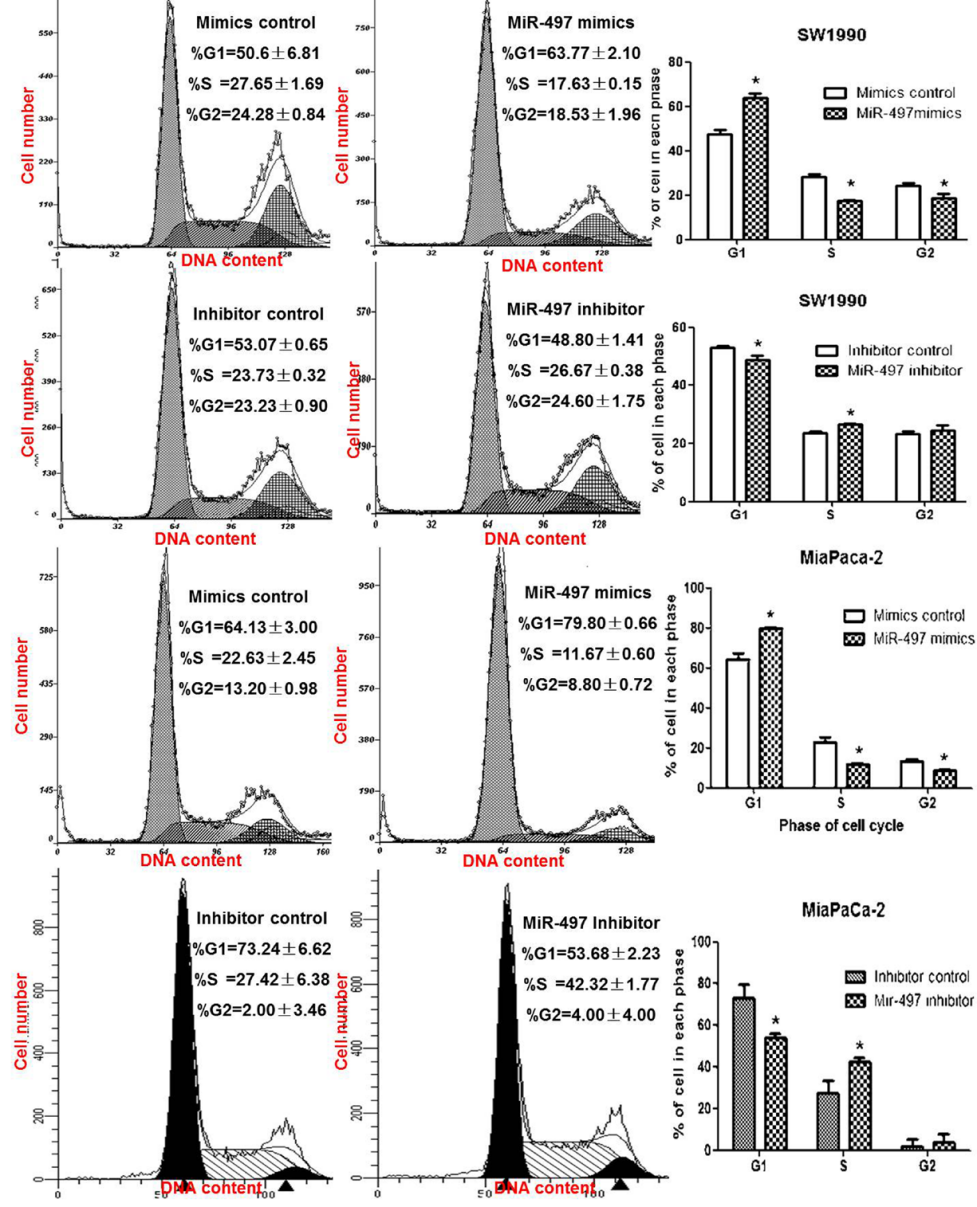
MiR-497 induced cell cycle arrest PDAC cells were transfected for 48 hours and collected for cell cycle analysis by fluorescence-activated cell sorting. Data are displayed as the mean ± SD (*P < 0.05).

We also evaluated the effect of miR-497 levels on cell cycle. Compared with the control group, upregulation of miR-497 in PDAC cells by transfection with mimics significantly decreased the percentage of cells in S phase (Figure 3), whereas knockdown of miR-497 by transfection inhibitors increased the percentage of S phase cells (Figure 3).

### MiR-497 attenuated the migration and invasion capacities

**Figure 4:**
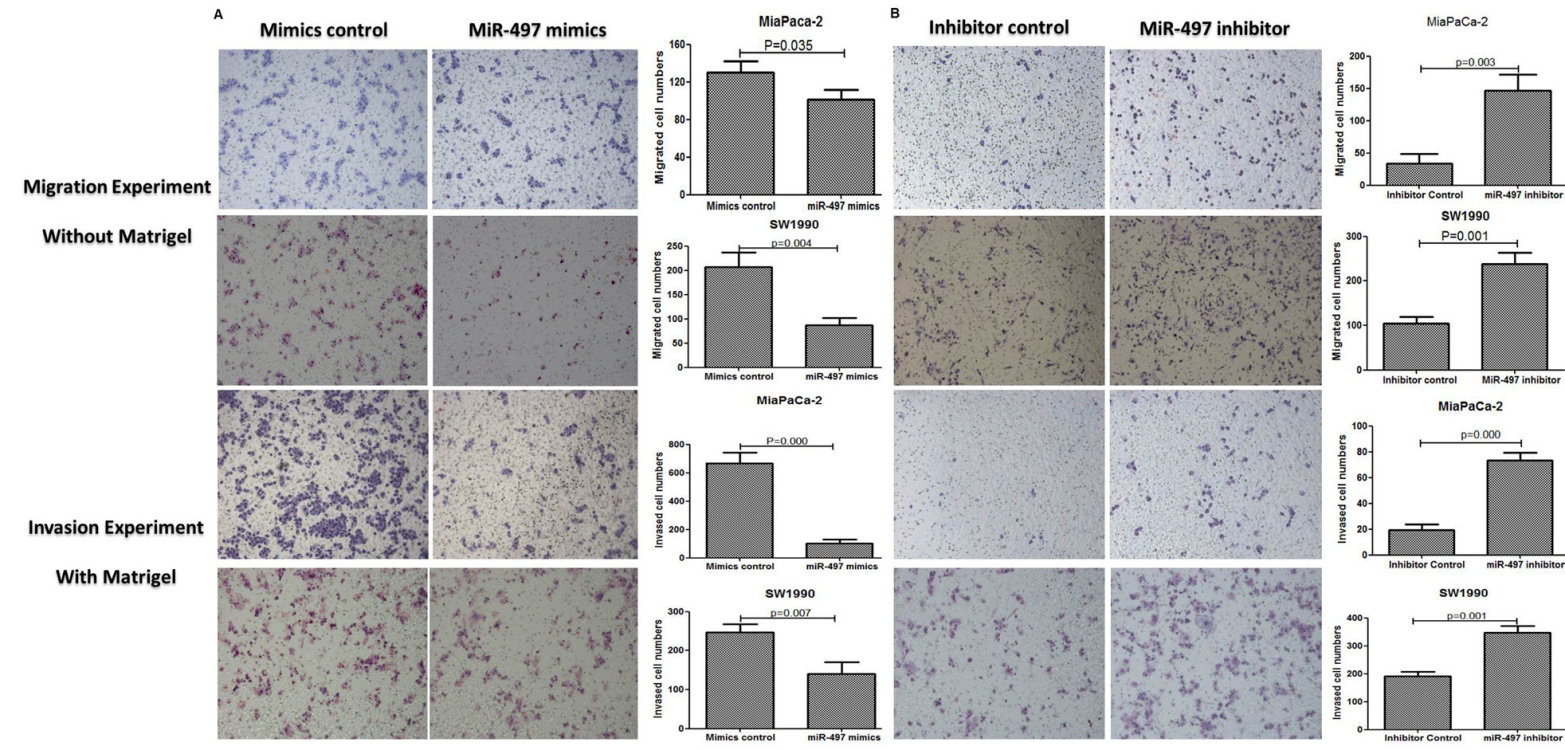
MiR-497 attenuated migration and invasion capacities Cell migration was analyzed by transwell membranes without Matrigel. Invasion was analyzed by transwell membranes with Matrigel. Cells that had migrated or invaded to the lower surface of the membrane were stained with hematoxylin and eosin and counted under a microscope at 100× magnification. (A) Upregulation of miR-497 by transfection with mimics reduced cell migration and invasion. (B) Downregulation of miR-497 by transfection of inhibitor promoted cell migration and invasion. Data are displayed as mean ± SD.

Transwell assays were performed to examine the contribution of miR-497 to cell migration and invasion in PDAC cell lines. We observed that upregulation of miR-497 in both PDAC cell lines resulted in reduced cell migration and invasion abilities (Figure 4A). In contrast, reduction of miR-497 levels enhanced cell migration and invasion (Figure 4B).

### MiR-497 inhibited tumor growth *in vivo*

**Figure 5:**
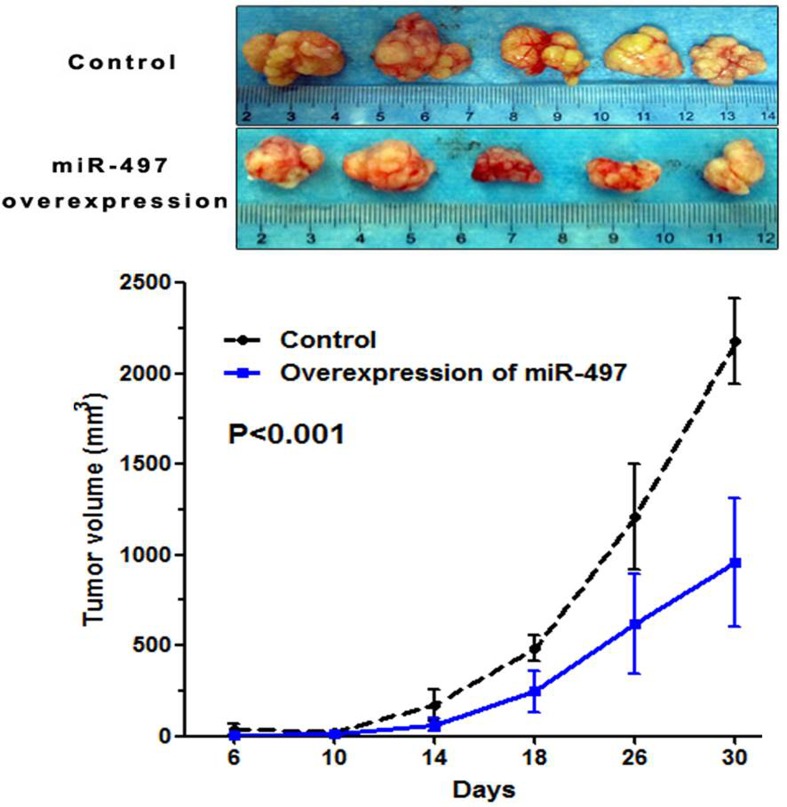
MiR-497 suppressed tumor growth *in vivo* Mice were transplanted with SW1990 cells stably overexpressing miR-497 or control cells, and tumor growth was monitored. Tumor growth *in vivo* was analyzed by analysis of variance. Upper panel: tumors from experimental and control mice. Lower panel: tumor growth curves showing miR-497 inhibition of tumor growth.

We next established an *in vivo* model to determine the role of miR-497 on tumor growth in mice. SW1990 cells stably overexpressing miR-497 or control cells were transplanted into mice, and tumor growth was monitored. Tumor size in mice transplanted with SW1990 cells stably overexpressing miR-497 was significantly smaller than those from control mice (*P*=0.000) (Figure 5).

### Identification of miR-497 targets

To identify potential targets of miR-497, we performed target prediction analysis using the bioinformatics database (TargetScan; http://www.targetscan.org/). Fibroblast growth factor 2 (FGF2) and fibroblast growth factor receptor 1 (FGFR1) were both identified as potential targets of miR-497.

**Figure 6:**
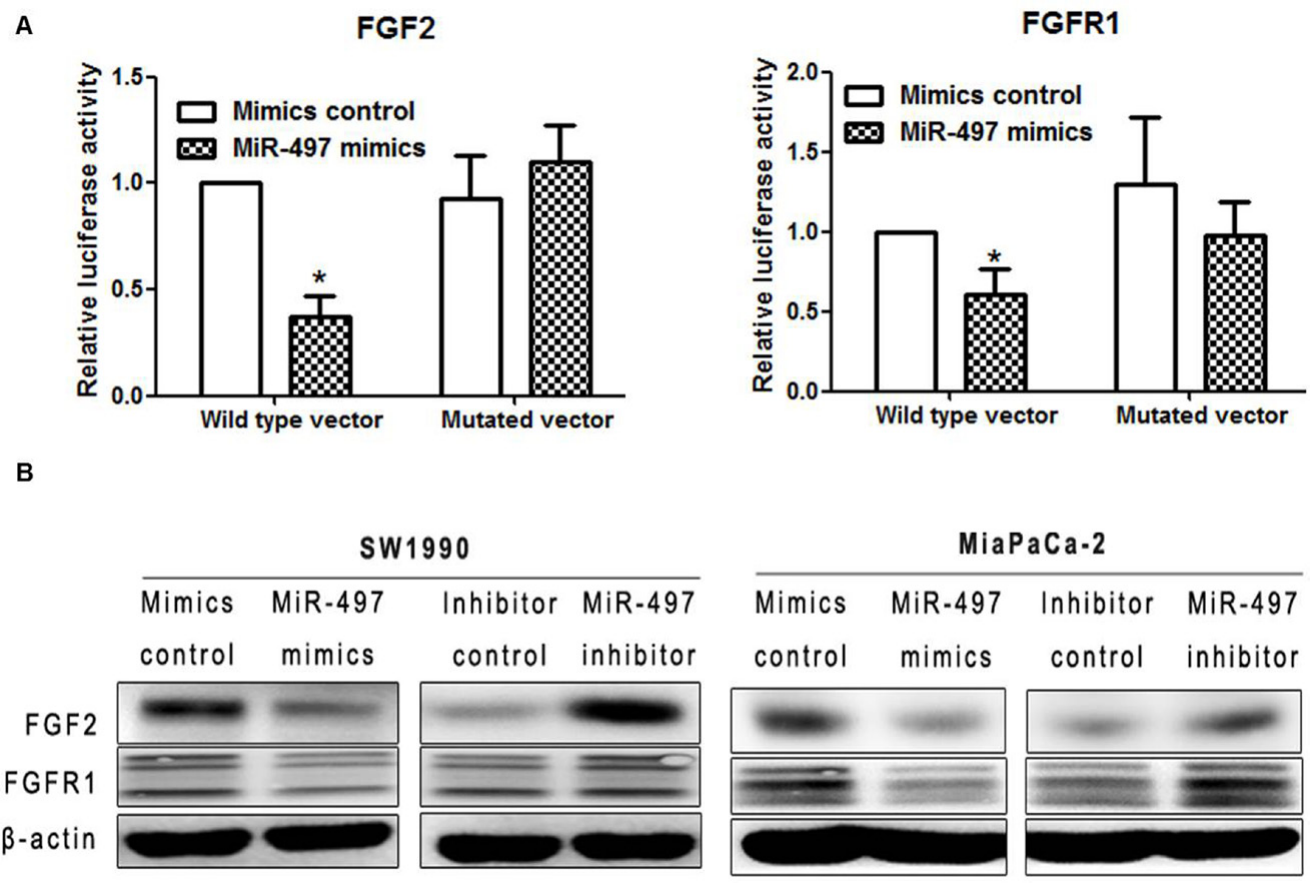
MiR-497 directly inhibited the expression of FGF2 and FGFR1 by binding to the 3′-UTR (A) Dual luciferase assay showed that FGF2 and FGFR1 were direct targets of miR-497. Cells were transfected with luciferase vectors containing either the wild-type or mutated binding site for miR-497 in FGF2 (left) or FGFR1 (right). The relative luciferase activity of cells co-transfected with vectors containing wild-type binding sequences along with miR-497 mimics was significantly decreased compared with that of cells co-transfected with mutated binding sequence vectors and mimics. Luciferase activities were also reduced compared with cells transfected with vectors containing wild-typed binding sequences and mimics control, as well as cells with mutant binding sequence vectors and mimics control. Data are displayed as the mean ± SD (*P < 0.05). (B) Protein expression levels of FGF2 and FGFR1 were detected by western blot.

We performed dual luciferase reporter assays for further verification. We generated luciferase reporter constructs containing either the wild-type or mutated binding sequences in FGF2 or FGFR1 downstream of the firefly luciferase gene. 293A cells were co-transfected with reporter vectors and mimics or mimic control, and luciferase activities were evaluated. Luciferase activities were significantly decreased after co-transfection of miR-497 mimics with vectors containing FGF2 and FGFR1 wild-type binding site sequences compared with cells co-transfected with mimics and vectors containing the mutated binding site sequence. Luciferase activities were also reduced compared with cells transfected with vectors containing wild-type binding sequences and mimics control, as well as cells with mutant binding sequence vectors and mimics control. (Figure 6A). These results suggest that FGF2 and FGFR1 are direct targets of miR-497.

To further confirm that FGF2 and FGFR1 are targets of miR-497, we performed western blot analysis. Upregulation of miR-497 significantly attenuated the expression of both FGF2 and FGFR1 protein levels, while inhibition of miR-497 increased the protein levels of FGF2 and FGFR1 (Figure 6B). Notably, upregulation of miR-497 had no effect on the mRNA levels of FGF2 and FGFR1 in SW1990 cells (Figure S2).

### MiR-497 downregulation in pancreatic cancer tissues

**Figure 7:**
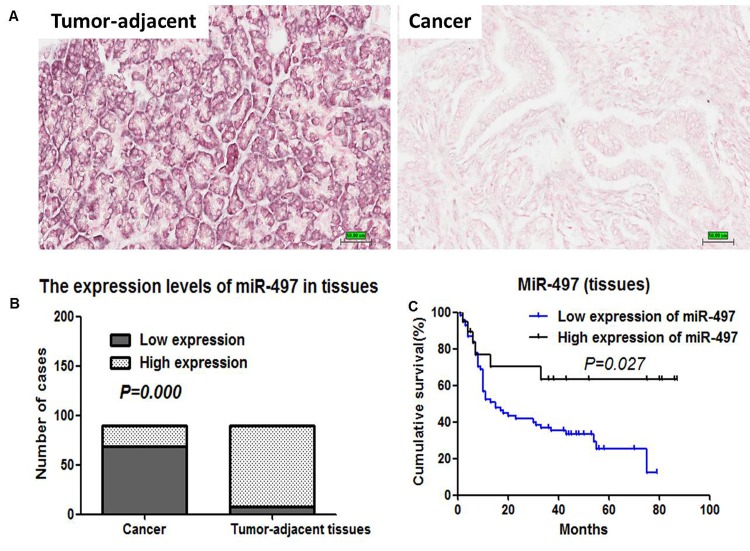
Expression levels and prognostic values of miR-497 in pancreatic cancer tissues (A) The expression levels of miR-497 in a panel of 90 pancreatic cancer and tumor-adjacent tissues were detected by ISH (200×). (B) The expression levels of miR-497 in 90 pancreatic cancer tissue samples and matched tumor-adjacent tissues were analyzed using Pearson χ^2^ test. (C) Kaplan-Meier survival analysis in pancreatic cancer patients based on miR-497 expression in primary tumors.

We next evaluated the expression levels of miR-497 in 90 pancreatic cancer tissue samples and matched tumor-adjacent tissues by in situ hybridization (ISH). ISH staining revealed that miR-497 was located in the cytoplasm (Figure 7A). Tissue samples were scored for high and low miR-497 expression, as described in Materials and Methods. Among the 90 pancreatic cancer samples, 69 showed low expression of miR-497 and 21 showed high expression. In comparison, among the matched tumor-adjacent tissues, only eight showed low expression of miR-497, whereas 82 showed high expression. The expression of miR-497 in pancreatic cancer tissues was significantly decreased compared with that in tumor-adjacent tissues (*P*=0.000) (Figure 7B).

### MiR-497 low expression was associated with a poor prognosis

We also assessed the correlation between miR-497 levels and clinicopathological parameters and prognosis. Three cases were lost to follow-up, and the statistical analysis was based on the remaining 87 cases. The mean follow-up was 26.9 months (range 1–87 months).

No correlation between the levels of miR-497 and clinicopathological parameters was observed (Table S1). Univariate survival analysis indicated that TNM staging and miR-497 expression levels were the potential prognostic factors of pancreatic cancer (Table 1, Figure 7C). Multivariate analysis demonstrated that TNM staging (II/III/IV) and miR-497 expression (low) were the independent adverse prognostic factors (*P*=0.016, hazard ratio [HR]=1.965, 95% confidence interval [CI]: 1.130–3.416, *P*=0.01, HR=2.762, 95%CI: 1.159–6.579, respectively).

**Table 1:** Univariate survival analysis

Parameters	Case number (n)	Median survival (Median±SE,months)	1-year survival rate (%)	*P* value
Sex				0.052
Male	56	13±3.5	81.9	
Female	31	54±11.0	63.6	
Age(years old)				0.693
<65	53	18±9.4	86.4	
≥65	34	15±16.1	69.8	
Locations				0.283
Head	53	23±12.1	61.8	
Body-tail	34	11±4.9	50.0	
Differential degree^a^				0.066
High/ moderate	55	33±12.8	66.3	
Low	23	10±1.5	43.6	
Tumor staging				0.833
T1/T2	72	17±8.1	54.8	
T3/T4	15	31±14.3	66.7	
Lymph node staging				0.061
N0	55	33±9.9	66.4	
N1	32	11±0.9	40.4	
TNM staging				0.027
I	43	37±13.8	68.6	
II/III/IV	44	11±2.7	45.6	
MiR-497 expression				0.027
Low	68	15±4.0	52.4	
High	19	None^b^	76.8	

a. The differential degree of 9 cases is not recorded.

b. None:If the number of censored data is more than 50% of the total, median survivals cannot be calculated by SPSS.

## DISCUSSION

Pancreatic cancer is a fatal disease, and difficulty in early detection and low resection rate may partly explain the poor prognosis. Another significant cause underlying poor prognosis is drug resistance. Thus, screening effective biomarkers, identification of the mechanisms of drug resistance and re-sensitizing pancreatic cancer cells to drugs are important strategies for improving overall survival. In the current study, we screened the miRNA profile associated with drug resistance in gemcitabine-resistant SW1990 pancreatic cancer cell lines, and identified 13 downregulated miRNAs and 20 upregulated miRNAs, including miR-497. We also found that miR-497 played important roles in regulating malignant phenotypes and drug resistance. In addition, we showed that the expression levels of miR-497 were significantly decreased in pancreatic cancer tissues and associated with a poor prognosis.

Previous studies have demonstrated that miRNAs are involved in chemoresistance to gemcitabine treatment in pancreatic cancer. However, most studies only focused on selected miRNAs [6, 9]. Only one study, performed by Iwagami et al., screened miRNAs associated with gemcitabine resistance in pancreatic cancer [10]. To well define the miRNAs associated with drug resistance, we screened the profile of miRNAs associated with drug resistance and identified 33 miRNAs that were dysregulated in gemcitabine-resistant SW1990 cell lines. Surprisingly, miRNAs identified in the present study were not identified in the report by Iwagami et al. [10]. Possible explanations include different cell lines used for experimental analysis and different selection criteria between these two studies.

Among the 33 miRNAs, we selected miR-497 for further study. We found that miR-497 suppressed proliferation, decreased the percentage of S phase cells, increased sensitivity to gemcitabine, and attenuated migration and invasion capacities, which was consistent with results from previous studies [8, 11]. Furthermore, we demonstrated for the first time that miR-497 increased the sensitivity of PDAC cells to erlotinib. Database prediction analysis identified potential targets of miR-497, including FGF2 and FGFR1. Dual luciferase reporter assays and western blot confirmed FGF2 and FGFR1 as direct targets of miR-497. Several members of the miR-15abc/16/16abc/195/322/424/497/1907 cluster had been identified to be involved in regulating the expression of FGF2 and FGFR1. MiR-15 and miR-16 were shown to inhibit the levels of FGF2 and FGFR1 protein by binding to the 3′-UTR [12]. However, our study provides the first findings indicating miR-497 regulation of FGF2 and FGFR1.

The FGF/FGFR signaling pathway is involved in multiple processes in cancer development. FGF/FGFR signaling activates several downstream signaling pathways, including the mitogen activated protein kinase and the phosphoinositide-3-kinase/AKT pathways [13, 14], which promote proliferation and metastasis and induce drug resistance and cell cycle progression.[15] Furthermore, activation of the AKT pathway results in acquired resistance to erlotinib treatment [16].

In addition, we found that the expression of miR-497 was significantly decreased in pancreatic cancer tissues. Our study further confirmed the results reported by previous studies [8, 17]. Survival analysis indicated that low miR-497 expression was an independent poor prognostic factor for pancreatic cancer patients. Similar results were reported in a study by Luo and colleagues demonstrating that low miR-497 expression appeared to be a poor prognostic factor in cervical cancer [17].

## CONCLUSION

The current study screened the miRNA profile associated with drug resistance in pancreatic cancer and identified a panel of miRNAs potentially involved in chemoresistance of pancreatic cancer cells. We demonstrated that miR-497 plays a role in modulating the malignant phenotype and chemosensitivity of pancreatic cancer cells by directly inhibition of FGF2 and FGFR1 expression. The expression levels of miR-497 were positively associated with overall survival, indicating that miR-497 could serve as a new prognostic biomarker in pancreatic cancer.

## METHODS

### Ethics Statement

Investigation has been conducted in accordance with the ethical standards and according to the Declaration of Helsinki and according to national and international guidelines and has been approved by the authors' institutional review board. All experiments involving animals and patients were approved by the Institutional Review Boards of Peking Union Medical College Hospital. Animal experiments were performed according to the guidelines of the Experimental Animals Management Committee. Written informed consent was obtained from all subjects.

### Cell lines and culture

SW1990 and MiaPaCa-2 pancreatic ductal adenocarcinoma (PDAC) cells (a gift from Professor Helmut Freiss of Heidelberg University, Germany) were cultured in a humidified incubator with 5% CO_2_ at 37°C in RPMI-1640 medium or Dulbecco's modified Eagle's medium (DMEM, Hyclone, Thermo Fisher Scientific Inc., Waltham, MA) containing 10% fetal bovine serum (FBS; Hyclone). 293A cell line is purchased from Cell Resource Center, IBMS, CAMS/PUMC, and cultured in DMEM containing 10% FBS. The gemcitabine resistant cell line, SW1990/GEM, was generated by intermittently increasing drug concentration. The initial concentration was the half maximal inhibitory concentration (IC50, 9 μg/ml). The drug concentration increased exponentially up to 1000 μg/ml over a period of 9 months. The IC50 of SW1900/GEM cells was 4560 μg/ml. Gemcitabine (750 ng/ml) was included in the medium to maintain the resistant phenotype and removed 1 month before experimental use of the cells.

### Illumina miRNA arrays

Total RNA was extracted from SW1990 and SW1990/GEM PDAC cells using TRIzol RNA isolation reagent (Invitrogen, Carlsbad, CA) according to the manufacturer's protocol. The Illumina Human miRNA Expression Profiling Version 2 Panels containing 1146 assays were used to screen the miRNA profile associated with chemoresistance of pancreatic cancer. Total RNA (200 ng) was polyadenylated by poly(A) polymerase and then reverse transcribed with biotinylated oligo-deoxythymidine primer with a universal PCR sequence at its 5′ end. The biotinylated cDNA was annealed to chimeric query oligonucleotides that included miRNA-specific oligos and a universal primer sequence for PCR amplification, and then bound to streptavidin-conjugated paramagnetic particles. After annealing, incorrectly bound or unbound oligos were removed by washing. DNA polymerase was added to extend the specific primer, and the resulting PCR products were purified. The labeled single-stranded products were hybridized to the beadchip overnight at 45°C. The beadchips were then scanned in a BeadArray Reader (Illumina) to obtain fluorescence intensity values. Data analysis was performed using the Gene Expression Module in Illumina GenomeStudio Software. Fold change > 4 or < 0.25 and *P* < 0.05 (Student *t* tests) were applied for selecting drug-resistant specific miRNAs.

### MiRNA transfection

For *in vitro* experiments, miRNAs at 50–100 nM were transfected using Lipofectamine 2000 transfection reagent (Invitrogen) according to the manufacturer's guidelines. MiR-497 mimics, mimics control, inhibitor and inhibitor control were synthesized by Genepharma (Shanghai, China). Sequences are listed as follows:

MiR-497 mimics 5′-CAGCAGCACACUGUGGUUUGU-3′
5′-AAACCACAGUGUGCUGCUGUU-3′
Mimics control 5′-UUCUCCGAACGUGUCACGUTT-3′
5′-ACGUGACACGUUCGGAGAATT-3′
MiR-497 inhibitor 5′-ACAAACCACAGUGUGCUGCUG-3′
Inhibitor control 5′-CAGUACUUUUGUGUAGUACAA-3′

### RNA isolation and quantitative RT-PCR (qRT-PCR) assay

Total cellular RNAs were isolated using TRIzol (Invitrogen) according to the manufacturer's protocol. For mRNA quantitative assay, total RNAs were reverse transcribed using the reverse transcription kit (Promega, Madison, WI) according to the manufacturer's guidelines. qRT-PCR was performed using the SYBR Green Master Mix (Takara, Japan). GAPDH was used as the internal control. For miRNA quantitative assay, TaqMan assays (Applied Biosystems) were performed according to the manufacturer's instructions. U6 served as an internal control. The levels of gene expression were calculated using the 2^−ΔΔCT^ methods. Primers are as follows:

FGF2 Forward primer 5′-AGTGTGTGCTAACCGTTACCT-3′
Reverse primer 5′-ACTGCCCAGTTCGTTTCAGTG-3′
FGFR1 Forward primer 5′-AATGAGTACGGCAGCATCAAC-3′
Reverse primer 5′-ACCTCGATGTGCTTTAGCCAC-3′
GAPDH Forward primer 5′-CGGAGTCAACGGATTTGGTCGTAT-3′
Reverse primer 5′-AGCCTTCTCCATGGTGGTGAAGAC-3′

### *In vitro* proliferation assay

PDAC cells were transfected in six-well plates (5×10^5^ cells/well) for 24 h, then trypsinized and reseeded in 96-well plates (1000 cells/well). Cell count kit (CCK-8) reagent (10 μl/well) was added at 0, 24, 48, 72 h and cells were incubated for 2.5 h at 37°C. Optical density (OD) was measured at the wavelength of 450 nm (OD450) by a microplate reader (Wellscan MK3, Thermo/Labsystems, Finland).

### Gemcitabine and erlotinib sensitivity assay

PDAC cells were transfected in six-well plates (5×10^5^ cells/well) for 24 h, then trypsinized and plated in 96-well plates (4000 cells/well). After another 24 h, cells were treated with different doses of gemcitabine (Eli Lilly and Company, Indianapolis, Indiana, USA) or erlotinib (Roche, Switzerland). After 48 h, 10 μl/well CCK-8 reagent was added, and cells were incubated for 2.5 h at 37°C. OD450 was measured by a microplate reader.

### Cell cycle assay

PDAC cells were transfected in six-well plates (5×10^5^ cells/well). After 48 h, cells were harvested, washed with cold phosphate-buffered saline (PBS) and fixed in 70% ethanol overnight at 4°C. After centrifugation with at 800–1000 rpm three times, cells were re-suspended with 500 μl PBS. Cells were stained with a solution containing 10 mg/ml RNase A, 0.1% Triton X-100 and 1 mg/ml propidium iodide. Cell cycle analysis was performed by fluorescence-activated cell sorting.

### Western blot

Cells were harvested 48 h after transfection in six-well plates and lysed with RIPA buffer (Applygen, Beijing). Total cell lysates (100 μg) were separated by sodium dodecyl sulfate polyacrylamide gel electrophoresis and transferred to a polyvinylidene difluoride membrane (Millipore, Billerica, MA). After blocking in 5% skim milk at room temperature for 1 h, the membranes were incubated with primary antibodies overnight at 4°C. The membranes were then probed with horseradish peroxidase-linked secondary antibodies at room temperature for 1 h and visualized using an echochemiluminescence detection system. Band intensities were quantified using Image-Pro Plus 6.0 software (Media Cybernetics, USA). Primary antibodies were as follows: anti-FGF2 antibody (Ab126861; Abcam), FGF Receptor 1 (D8E4) XP® Rabbit mAb (9740; Cell Signaling Technology), and β-Actin (13E5) Rabbit mAb (4970; Cell Signaling Technology).

### Transwell assay

Cell migration and invasion were assessed by transwell assay. Cell migration was analyzed by transwell membranes without Matrigel. Invasion was analyzed by transwell membranes with Matrigel. Transfected cells were seeded in medium without FBS in the upper chamber of a transwell system (polycarbonate membrane, diameter 6.5 mm, pore size 8 μm; Corning Costar, USA). Media with 10% FBS was added into the bottom chamber. After 24–48 h incubation (SW1990: 24 h; MiaPaCa-2: 48 h), migrated or invading cells were fixed with formalin and stained with hematoxylin and eosin (Zhong Shan Golden Bridge Company, Beijing, China). Cell numbers were counted under a microscope at 100× magnification in five random visual fields. Experiments were performed three times.

### Animal experiments

SW1990 PDAC cells stably transfected with miR-497-lentiviral vectors or control lentiviral vectors were injected subcutaneously into the right back of 6-week-old female BALB/c mice (3×10^6^ cells in 200 μl PBS per mouse). Each experimental group included five mice. Tumor size was measured twice a week using caliper measurement of two perpendicular diameters of the implants. Tumor volume (mm^3^) was calculated based on the formula: volume (mm^3^) = 1/2 × length × width^2^. The tumor-bearing mice were euthanized on the 30^th^ day.

### Luciferase reporter assays

The pmirGLO dual luciferase miRNA target expression vector (Promega, E1330) was used to assess miR-497 regulation of putative miRNA target sites. Wild-type or mutant miR-497 binding site sequences in FGF2 and FGFR1 were synthesized by Invitrogen and cloned into pmirGLO vectors 3′ of the firefly luciferase gene. Vectors were named as wild-type and mutated vectors. Vectors and miR-497 mimics or mimic control were co-transfected in 293A cells in 12-well plates (1×10^5^ cells/well) using Lipofectamine 2000 reagent. After 48 h, luciferase activities were evaluated using the Dual-Luciferase Reporter Assay System (Promega) according to the manufacturer's guidelines. Renilla luciferase (hRluc-neo) served as the control reporter for normalization.

### Pancreatic tissue collection and in situ hybridization (ISH)

Formalin-fixed, paraffin-embedded pancreatic cancer tissues (n=90) and matched tumor-adjacent tissues (n=90) were collected from Peking Union Medical College Hospital and used to create tissue microarrays. MiRCURY LNA™ detection probe (38256-15; Exiqon, Vedbaek, Denmark) was used to detect the expression levels of miR-497 in tissues. For ISH, slides were incubated at 37°C for 30 min. After slides were deparaffinized in xylene and rehydrated with graded alcohol washes, the slides were fixed in 4% paraformaldehyde for 20 min and then washed in PBS three times. The slides were then incubated with 15 μg/ml proteinase K for 15 min at room temperature. Slides were washed in PBS and fixed in 4% paraformaldehyde for 15 min. After rinsing in PBS, slides were pre-hybridized in hybridization buffer for 1 h at 50°C and then hybridized in the hybridization buffer containing probe overnight at 4°C. The following day, slides were washed stringently at 50°C for 20 min and then blocked in a blocking solution for 1 h at room temperature. Finally, slides were placed in blocking solution containing alkaline phosphatase conjugated anti-DIG Fab fragment overnight at 4°C. The colorimetric detection reaction was carried out using the NBT/BCIP kit (ThermoFisher Scientific) according to the manufacturer's introductions.

Slides were scored according to the staining intensity and number of positive cells. Scoring for staining intensity was as follows: none (0 point), weak staining (1 points), intermediate staining (2 points), and strong staining (3 points). Scoring for the percentage of positive cells was as follows: absent (0 point), 1–24% positive cells (1 point), 25–49% (2 points), 50–74% (3 points), and 75–100% (4 points). The final score was calculated by multiplication of the above two scores. MiR-497 expression was considered to be low if the final score was less than 4 points, and high if the final score was 4 or more points [18].

Follow-up data were obtained from hospital records supplemented with telephone contact. The end point was overall survival. Survival time was defined according to the date of death or as the time between the last follow-up date and the operation date.

### Statistical analysis

Statistical analysis and graph presentation were performed using SPSS v.13.0 software (SPSS Inc., Chicago, IL) and GraphPad Prism 5 Software (GraphPad, San Diego, CA), respectively. Measurement data were presented as mean ± standard deviation (SD) and compared by Student's *t* test or the Mann-Whitney *U* test. Categorical data were compared by Pearson χ^2^ test or Fisher exact test. The Kaplan–Meier method and Cox regression were used for univariate and multivariate survival analysis. A value of *P* < 0.05 was considered as statistically significant.

## SUPPLEMENTARY MATERIAL FIGURES AND TABLE


