# Supplementary material for: MiR-497 downregulation contributes to the malignancy of pancreatic cancer and associates with a poor prognosis
Source: Oncotarget. 2014 Jul 8;5(16):6983–93. doi: 10.18632/oncotarget.2184 (PMC4196178; doi:10.18632/oncotarget.2184)
Supplement: Supplementary file 1 [file oncotarget-05-6983-s001.docx]

MiR-497 downregulation contributes to the malignancy of pancreatic cancer and associates with a poor prognosis

**Figure S1: Expression levels of miR-497 in cells transfected with mimics or inhibitor.** SW1990 cells were transfected with mimics, inhibitor or controls for 48 h. Total RNA was isolated and the expression levels of miR-497 were detected by qRT-PCR. U6 was used as an internal control. Data are displayed as the mean ± SD (**P* < 0.05).

**Figure S2: Expression levels of FGF2 and FGFR1 mRNA in transfected cells.** SW1990 cells were transfected with mimics or controls for 48 h. Total RNA was isolated and the expression levels of mRNA were detected by qRT-PCR. GAPDH was used as an internal control. Data are displayed as the mean ± SD (**P* < 0.05).

**Table S1: Correlations of miR-497 levels and clinicopathological parameters**

| **Parameters** | **Low group**  **(n=68)** | **High group**  **(n=19)** | ***P* value** |
| --- | --- | --- | --- |
| **Gender** |  |  | 0.337 |
| Male | 42 | 14 |  |
| Female | 26 | 5 |  |
| **Age（years old）** |  |  | 0.76 |
| ＜65 | 42 | 11 |  |
| ≥65 | 26 | 8 |  |
| **Locations** |  |  | 0.448 |
| Head | 40 | 13 |  |
| Body-tail | 28 | 6 |  |
| **Differential degree ^a^** |  |  | 0.540 |
| High/moderate | 45 | 10 |  |
| Low | 17 | 6 |  |
| **Tumor staging** |  |  | 1.000 |
| T1/T2 | 56 | 16 |  |
| T3/T4 | 12 | 3 |  |
| **Lymph node staging** |  |  | 0.586 |
| N0 | 44 | 11 |  |
| N1 | 24 | 8 |  |
| **TNM staging** |  |  | 0.470 |
| Ⅰ | 35 | 8 |  |
| Ⅱ/Ⅲ/Ⅳ | 33 | 11 |  |

a.The differential degree of 9 patients is not defined.
